# Education in Health

**Published:** 1924-10

**Authors:** 


### EDUCATION IN HEALTH.

"There has been a rapid development of the whole system of sanitary governance and public medical services, and public opinion has not kept pace with these two profound changes." So writes Sir George Newman, the Chief Medical Officer, in the admirable Memorandum on "Public Education in Health" which he has just addressed to the Minister of Health. It is the old story of the need for the governors to educate the governed. Put into a sentence, Sir George Newman's thesis, stated incisively and in excellent literary form, is that neither the local health authorities nor individual citizens are doing their full duty in seeking health and ensuing it. The local authorities do much, but they might, and ought to, do more; the many voluntary organisations which have accomplished such great things for motherhood and child welfare, in combating tuberculosis and securing clean milk, and in a host of other directions, might be more closely associated with them, and the work of the two brought more definitely into line. The sanitary administration of the country, taken all round, is of first-rate quality; what is now needed is that the individual should be brought to see that the prevention of disease is his personal concern rather than a matter exclusively dependent upon either the central or the local health authorities. Administration is one thing; health education and propaganda are another. The Ministry of Health and the Board of Education have both developed remarkable activity in their educative work. The local authorities have, in the main, done what they could, but their powers of expenditure in this direction are limited.

As the pioneer of sanitation and the earliest teacher of hygiene, we have to maintain, and increase, a reputation which has hardly kept pace with the incessant activity of the United States. This is no mere question of "Baby Weeks" and the like, excellent and desirable as such things are. As Sir George Newman warns us, we must "seek to get the whole business of the education of the public in these matters into its true perspective in relation to the recognised methods of sanitary government in England." The people must be sufficiently educated to bring compulsion to bear upon authority, and in the process of that education they will learn that each man is the custodian of his own personal health, and, as such, responsible both to himself and the community, both of which he has power to injure or to benefit. Sir George Newman would so amend the law that there may no longer be any doubts about the educational functions of local authorities, and that there should be imposed upon all of them "a power or a duty of direct educational work." We may then have to consider the desirability, and perhaps the necessity, of assisting such education by an Exchequer grant. These methods of instruction are numerous and some are better suited to a given district than others. But, whatever the plans that are adopted, it is essential that they should all be directed to bringing home to the ignorant or the merely thoughtless a clear perception of the fact that, although health is the gift of God, the loss of it is not necessarily the work of the devil. Most people commit suicide, without knowing or intending it.

